# Performance optimization of the Varian aS500 EPID system

**DOI:** 10.1120/jacmp.v7i1.2158

**Published:** 2006-02-21

**Authors:** Lucie Berger, Pascal François, Geneviève Gaboriaud, Jean‐Claude Rosenwald

**Affiliations:** ^1^ Medical Physics Department Institut Curie 26 rue D'Ulm Paris F‐75005 France

**Keywords:** electronic portal imaging device, acquisition parameters, dose, image quality

## Abstract

Today, electronic portal imaging devices (EPIDs) are widely used as a replacement to portal films for patient position verification, but the image quality is not always optimal. The general aim of this study was to optimize the acquisition parameters of an amorphous silicon EPID commercially available for clinical use in radiation therapy with the view to avoid saturation of the system. Special attention was paid to selection of the parameter corresponding to the number of rows acquired between accelerator pulses (NRP) for various beam energies and dose rates. The image acquisition system (IAS2) has been studied, and portal image acquisition was found to be strongly dependent on the accelerator pulse frequency. This frequency is set for each “energy — dose rate” combination of the linear accelerator. For all combinations, the image acquisition parameters were systematically changed to determine their influence on the performances of the Varian aS500 EPID system. New parameters such as the maximum number of rows (MNR) and the number of pulses per frame (NPF) were introduced to explain portal image acquisition theory. Theoretical and experimental values of MNR and NPF were compared, and they were in good agreement. Other results showed that NRP had a major influence on detector saturation and dose per image. A rule of thumb was established to determine the optimum NRP value to be used. This practical application was illustrated by a clinical example in which the saturation of the aSi EPID was avoided by NRP optimization. Moreover, an additional study showed that image quality was relatively insensitive to this parameter.

PACS numbers: 87.53.Oq; 87.59.Jq

## I. INTRODUCTION

Portal imaging is currently used to verify patient position during radiation treatment.^(^ ^1^ ^–^ ^4^ ^)^ A specific subject of interest with electronic portal imaging devices (EPIDs) is their ability to determine patient dose. Portal imaging systems are therefore developed to provide both geometrical and dosimetric information.^(^ ^5^ ^–^ ^8^ ^)^ Compared to previous systems, the amorphous silicon‐based EPID provides better quality portal images,^(^ ^9^ ^,^ ^10^ ^)^ but in some cases with a high dose rate or short source‐to‐detector distance (SDD), saturation can occur.^(11)^ This saturation can be avoided by optimizing the software acquisition parameters.

This study assessed the performance of the aS500 Varian (Palo Alto, CA) EPID. The influence on portal image acquisition and dose per image of a major acquisition parameter, the number of rows acquired between two consecutive accelerator pulses, called the number of rows/pulses (NRP), was studied. The influence of this parameter on the EPID response was investigated as a function of the accelerator pulse rate repetition. Subsequently, the influence of NRP and of the SDD on the detector saturation was assessed.

## II. MATERIALS AND METHODS

### A. The aSi‐based EPID

EPID measurements were performed using an aSi portal imager (aS500, Varian Medical Systems) in service mode. This system includes the following components: (1) image detection unit (IDU), featuring the amorphous silicon (aSi) detector and accessory electronics; (2) image acquisition system 2 (IAS 2) containing acquisition electronics for the IDU and interfacing hardware; and (3) a PortalVision workstation. Within the detector, a scintillator converts the incoming X‐rays into visible photons. The light is sensed by a photodiode array attached to the amorphous silicon panel. The photodiodes integrate the incoming light into charge captures, and the detector electronics transfer the charges from pixels to read‐out electronics. The sensitive area of the panel is 512×384 pixels, with a pixel size of 0.784 mm.^(12)^ An image of almost 200 000 pixels is obtained by activating the pixel matrix row after row. The number of rows read between two pulses is controlled by the NRP parameter, which can be chosen by the user.

### B. Portal image acquisition theory

**Figure 1:**
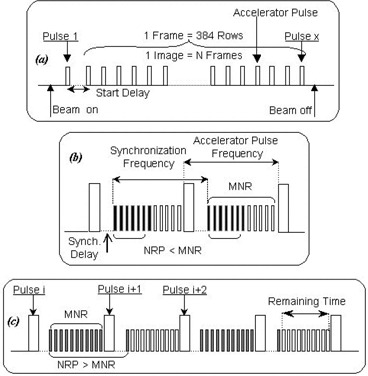
Portal image acquisition theory. One frame consists of 384 rows read between accelerators pulses. The Start Delay (a) defines the delay time between switching the beam on and starting the image acquisition (beam stabilization time). The Synch. Delay δ (b) is the waiting time after a beam pulse has occurred before the start of row scanning. Two cases, NRP<MNR and NRP>MNR, are displayed on Figs. 1(b). and 1(c), respectively. Black and white bars represent the rows actually read and potentially readable before the next beam pulse, respectively.

The entire matrix is read row by row. Rows are read between beam pulses (Fig. 1(a)). The NRP parameter defines the total number of rows to be read by the IAS2 before charges are transferred from pixels to read‐out electronics. The pulse frequency determines the interval between two pulses.

From Fig. 1(b) it is clear that the maximum number of rows (MNR), which can be acquired between two pulses, can be calculated using the expression

(1) $$\text{MNR}=\left(\frac{1}{v}-\delta \right)/T_{r}$$

where ν is the accelerator pulse frequency, δ is the synchronization delay (Synch.Delay), that is, the waiting time after a beam pulse before the start of row scanning (see Fig. 1(b)), δ=1ms, and $T_{r}$, the row acquisition time, is 0.203 ms.

In what follows, as a simplification, the term “dose” will be used to describe the aSi detector response.

The dose per frame (DF) measured by the aSi detector corresponds to the dose deposited by the number of pulses delivered during the interval between two readings of the same row, that is, the number of pulses per frame (NPF).

NPF is calculated by expression (2):

(2) $$\text{NPF}=\frac{T_{i}}{N}\times v=T_{f}\times v$$

where $T_{i}$ is the image acquisition time, its value obtained from the image file properties; *N* is the number of averaged frames per image; its value is chosen by the user and was 4 in this study; ν is the accelerator pulse frequency; and $T_{f}=T_{i}/N$ is the frame acquisition time.

DF is obtained by multiplying NPF by the dose per pulse. DF may therefore be calculated using the following expression:

(3) $$\text{DF}=\text{NPF}\times \frac{\dot{D}}{v}$$

where Ḋ is the “dose” rate, that is, detector response/second.

In the acquisition software, NRP is set by the user. There is no software restriction, and an NRP larger than the maximum number of rows between consecutive pulses (MNR) can be set. Two cases are discussed.

#### (1) NRP<MNR (see Fig. 1(b))

An entire matrix consists of 384 rows, and all rows are read between the accelerator pulses. As NRP increases, more and more rows are acquired for the same number of pulses, resulting in a greater efficacy and a lower DF. Thus, when the number of rows read between consecutive pulses increases, DF measured by the detector is expected to be lower.

#### (2) NRP>MNR (see Fig. 1(c))

Pulse *i*: MNR is read during the interval between pulse *i* and pulse i+1. The number of rows (NRP value) is not completely read during this time interval.

Pulse i+1: The IAS2 continues to read up to the number of rows, NRP, which is set by the user. When all rows have been read, the IAS2 stops reading until the next radiation pulse, which leaves a spare interval during which no rows are acquired, resulting in a longer acquisition time to read the entire image.

From the above equations it appears that the NPF variation that is proportional to the EPID response (DF) may be obtained experimentally.

### C. Measurements

Four X‐ray beam energies ranging from 4 MV to 20 MV were investigated on two Varian LINACS (2300 EX and 2100 C/S). The detector was placed 140 cm from the source, and the full detector surface was irradiated. The SDD was 140 cm for all measurements except when otherwise indicated. Averaging signals of a definite number of frames, called “average frames,” create the portal image. This number of frames depends on the acquisition mode selected. Typically, a portal image is obtained from averaging either 4 frames (low‐quality mode) or 10 frames (high‐quality mode). The frame acquisition is synchronized with accelerator beam pulses. If the frame acquisition starts at the first beam pulse, immediately after the beam is on, there may be one or two initial frames influenced by the beam stabilization. In order to improve the image quality, these initial frames should be discarded. They are called “reset frames.” For our measurements, the images consisted of two reset frames, for ensuring beam stability, and four average frames, for acceptable statistics. The EPID response was assessed by averaging 10×10 pixels in a squared region of interest centered on the beam axis and multiplying this value by the acquisition time. Energy and frequency dependence were studied. The influence of NRP was investigated with respect to the detector saturation, the dose per image, and the SDD. The aSi detector was calibrated (dark field and flood field corrections) before each modification of the NRP parameter.^(13)^ Our results were confirmed and illustrated by a practical example and a clinical application.

## III. RESULTS

### A. Energy and frequency dependence

For the four X‐ray beam energies studied in this work, different monitor unit (MU) rates may be selected ranging from 100 MU/min to 400 MU/min for the 6‐ and 20‐MV X‐ray beams, from 50 MU/min to 250 MU/min for 4 MV, and from 80 MU/min to 400 MU/min for the 10 MV beam. The Varian LINACs use a reference synchronization frequency different for each X‐ray beam energy, which corresponds to a maximum theoretical dose rate (see Table 1). The LINAC dose rate is varied by keeping only a fraction (one‐sixth) of these pulses (1 of 6, 2 of 6, etc.), but the pulse sequence could then be irregular (e.g., 5 of 6). This pattern serves as a basis to generate regular pulses required for portal imaging providing an acquisition pulse rate repetition according to Table 2. This acquisition pulse frequency is used to calculate MNR (see Eq. (1)) and has a direct influence on NPF variations (i.e., dose variations).

**Table 1:** Reference synchronization pulse frequencies for the beams used in this work

	Clinac 2100 C/S		Clinac 2300 FX	
energy (MV)	4	10	6	20
synchronization frequency (Hz)	400	360	360	180

**Table 2:** Acquisition pulse frequencies for each experimental condition in this work

	Clinac 2100C/S				Clinac 2300 EX		
4MV		10 MV		6 MV		20 MV	
MU rate (MU/min)	Freq.(Hz)	MU rate (MU/min)	Freq.(Hz)	MU rate (MU/min)	Freq.(Hz)	MU rate (MU/min)	Freq.(Hz)
50	66.67	80	60	100	60	100	30
100	133.33	160	120	200	120	200	60
150	200	240	180	300	180	300	90
200	133.33	320	120	400	240	400	120
250	66.67	400	60				

**Figure 2:**
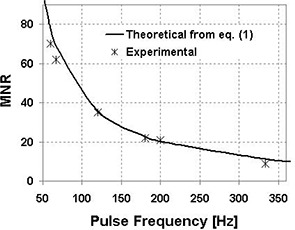
Theoretical and experimental MNR as a function of acquisition pulse frequency. The theoretical data are calculated using Eq. (1) with a synchronization delay δ=1 ms and a row acquisition time $T_{r}=0.203\;\text{ms}$.

Figure 2 represents the MNR variations as a function of the acquisition pulse frequency. When the frequency increases, MNR becomes smaller. The theoretical data are calculated from Eq. (1), and the experimental data are derived from the EPID response variation (see below Fig. 4), by looking at the NRP difference between two consecutive minima. There is good agreement between theory and experiment, considering that discrepancies could result from δ uncertainties in Eq. (1) and also from uncertainties on measured NPF. This uncertainty is approximately 2%.

### B. Saturation

Saturation may happen during the A/D conversion. The 14‐bit A/D component converts the analog signal from the pre‐amplifier into a signed 13‐bit value, that is, $8192\;(2^{13})$. Thus, when pixel counts exceed an absolute value of 8192, saturation can occur,^(11)^ especially for a high MU rate (400 MU/min) and a short SDD (e.g., 105 cm).

**Figure 3:**
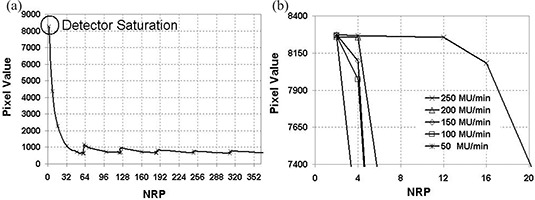
(a). Averaged central pixel value as a function of NRP for a 4‐MV beam. (b) Influence of the MU rate on pixel value for a 4‐MV beam for a MU rate of 250 MU/min and a SDD=140cm. Detector saturation is observed for NRP ≤16.

NRP has a major influence on detector saturation. When the accelerator dose rate increases or when the SDD decreases, DF increases. Consequently, the absolute value of the pixel count also increases. To avoid saturation, a large NRP value must be set (see Fig. 3(a)). Figure 3(b) displays the average pixel value in the central area as a function of NRP for the various MU rates of the 4‐MV beam. When the dose rate increases, a larger NRP must be set to avoid saturation.

### C. Dose per frame

**Figure 4:**
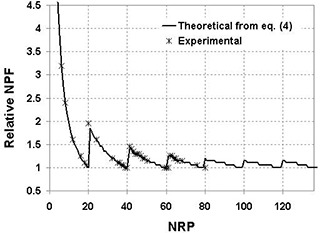
Theoretical and experimental relative NPF (i.e., relative dose) normalized to its minimum (at NRP=20) as a function of NRP for a 4‐MV beam with a MU rate of 150 MU/min. The theoretical data are calculated using Eq. (4) with an accelerator pulse frequency of 200 Hz (Table 2.

DF is directly proportional to NPF (see Eq. (3)). NPF undergoes a major relative variation as a function of NRP. Figure 4 shows that, when NRP increases, the relative NPF, that is, the relative dose per frame, decreases to a minimum corresponding to MNR, the maximum number of rows that can be acquired per pulse. As NRP becomes greater than MNR, more pulses are needed to read the entire matrix. NPF reaches a peak and then decreases until a second minimum corresponding to twice MNR, etc. Discontinuities are expected. The experimental relative NPF curve may be described by a theoretical expression (see Eq. (4)), obtained from an intuitive “trial and error” approach, that explains the variations displayed in Fig. 4.

(4) $$\text{NPF}=\text{INT}\left(\frac{n}{\text{NRP}}\right)\times \left[\text{INT}\left(\frac{\text{NRP}-1}{\text{MNR}}\right)+1\right]+\text{INT}\left[\frac{\left(\text{MOD}\left(\frac{n}{\text{NRP}}\right)+\text{MNR}-1\right)}{\text{MNR}}\right]$$

where $\text{INT}\left(\frac{a}{b}\right)$, is the integer part of the result of the division of $\left(\frac{a}{b}\right),\;\text{MOD}\left(\frac{a}{b}\right)$, is the remainder of the division of $\left(\frac{a}{b}\right),\;n$, the total number of rows of the entire matrix, is 384 rows, *MNR* is calculated using Eq. (1), and $\text{INT}\left(\frac{n}{\text{NRP}}\right)$ is the number of charge transfers required to read the 384 rows of the matrix. This first term describes the decreasing part of the curve, and $\text{INT}\left(\frac{\text{NRP}-1}{\text{MNR}}\right)+1$ represents the number of accelerator pulses necessary to read the number of rows chosen by the user (NRP) before transferring the charges from pixels to read‐out electronics. It explains the discontinuities.

$\text{INT}\left[\frac{\left(\text{MOD}\left(\frac{n}{\text{NRP}}\right)+\text{MNR}-1\right)}{\text{MNR}}\right]$ is the number of pulses necessary to read the remaining rows.

For a 4‐MV beam with a MU rate of 50 MU/min (energy and MU rate used for portal images in routine clinical practice), DF may vary by a factor of 50, depending on the NRP value selected.

### D. Source‐to‐detector distance dependence

When the SDD decreases, the dose rate increases and frequency remains unchanged. Saturation is avoided if the user sets a larger NRP. The detector response was measured at two different SDDs: 105 cm and 140 cm. An NRP value larger than 8 was necessary to avoid saturation at 105 cm, whereas NRP>6 was sufficient at 140 cm. It should also be noted that when saturation is avoided, the detector response variation follows the inverse square law.

### E. Practical application

In our institution, we have established a rule of thumb to quickly determine the optimum NRP value to be used. Experimental relative dose curves were used, and the following rules have been set to obtain Eqs. (5) and (6) below:

1. If NRP>MNR, NPF should not be in the initial part of the curve where the gradient is very high (Fig. 4),
2. Depending on the pulse frequency, two cases were considered:
  - for low frequencies (<70Hz), the NRP value is set just after the second minimum: NRP=2MNR+1/3MNR (see Eq. (5)),
  - for higher frequencies (>70Hz), it is set just after the fourth minimum: NRP=4MNR+1/2MNR (see Eq. (6)).
3. NRP≠(MNR+1),NPF, NPF should not be on a peak of the curve.

The maximum efficiency (without alteration of image quality) in terms of patient dose optimization and absence of saturation is therefore obtained from the following empirical expressions:

Pulse frequency <70Hz:

(5) NRP=2.33×MNR

Pulse frequency >70Hz:

(6) NRP=4.5×MNR

where NRP corresponds to the number of rows read between two pulses, and MNR is the maximum number of rows that can be acquired per pulse obtained from Eq. (1); pulse frequency is given in Table 2.

### F. Clinical example: Dose versus field size

The “dose” measured by the aSi detector on the beam axis as a function of field size was studied. The results were normalized to a field size of 10cm×10cm.

For the measurements, a 10‐MV beam with the maximum clinical MU rate (400 MU/min) was used. The detector was placed as close as possible to the source, which corresponds to a SDD of 105 cm. No attenuator was placed between the source and the aSi detector. Field sizes ranged from 5cm×5cm to 27cm×27cm. Images were obtained with 100 MU.

**Figure 5:**
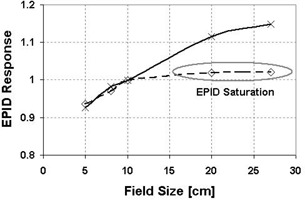
Variation of the aSi EPID response on the beam axis with the field size. The solid and dashed lines represent aSi response with and without optimization of the NRP parameter, respectively.

Figure 5 displays the aSi measurements with and without optimization. Before optimization, detector saturation occurred for large field sizes. To avoid detector saturation, an optimized NRP value was determined, and the aSi detector was able to measure a higher dose without saturation. Neither an additional attenuator nor a dose rate reduction was necessary to perform reliable acquisition.^(11)^

## IV. DISCUSSION

The use of EPID in radiation therapy could lead to portal images of poor quality if inconsistent acquisition parameters are selected. Saturation of the aSi EPID is due to the A/D conversion. The limits beyond which saturation starts to occur have been investigated by Van Esch et al.^(11)^ They have shown that for a short SDD, saturation effects are expected for all acquisitions, except for the low dose rate settings. Moreover, for the highest dose rates, saturation becomes an issue even in the standard frame acquisition mode. Other authors have studied the performance and the limitation of the aSi EPID.^(^ ^10^ ^,^ ^14^ ^,^ ^15^ ^)^ Vetterli et al. have even developed a new acquisition mode for PortalVision™ aS500 EPID, which allows one to take portal images with reduced dose while keeping good image quality.^(16)^ But the saturation problem and the optimization of acquisition parameter were never explicitly treated.

The image acquisition system (IAS2) was studied, and EPID response was found to be dependent on the accelerator pulse frequency. This frequency is dependent on each “energy‐dose rate” combination of the linear accelerator. The influence of the critical parameter NRP on the performances of the Varian aS500 EPID system has been evaluated. NRP has a major influence on detector saturation. We have shown that NRP optimization could allow one to avoid this saturation. A simple rule has been established to determine the optimum value of NRP. This rule depends on another parameter, the maximum number of rows that can be acquired between two accelerator pulses, which is fixed according to the accelerator pulse frequency.

When such acquisition parameters are changed, the image quality should be unchanged. This has been checked by an additional study. The NRP influence on image quality was assessed by measurements of noise, homogeneity (both horizontal and vertical), spatial resolution, and contrast performed using a contrast‐detail phantom (Las Vegas phantom, Varian). The image quality indexes were relatively insensitive to NRP. As expected, no particular tendency was observed. However, we observed that when NRP was set exactly to MNR+1, that is, corresponding to the peaks of the dose response curve, the quality was poorer with regularly spaced strips seen on the image.

NRP and all other acquisition parameters could be changed in service mode. NRP should be optimized with Eq. (5) or Eq. (6). The change of NRP value will affect portal image acquisition in clinical mode so an image calibration should be redone after every change.

This study is restricted to the aS500 Varian EPID and to the image acquisition principle on Varian LINACs. The results would probably be quite different for other accelerators and/or EPIDs, where similar studies should be performed. In electron beams, where the *bremsstrahlung* component may be used for image formation,^(^ ^17^ ^–^ ^19^ ^)^ the pulse rate pattern is not necessarily similar to the one used for photon beams, and the conclusions may also be quite different.

## V. CONCLUSION

The acquisition parameters of the aS500 EPID have been studied, and NRP has been optimized for clinical use in radiation therapy. A predictive model of EPID response as a function of these parameters has been established. The DF, which is proportional to NPF, exhibits strong and irregular variation as a function of NRP. This variation is strongly dependent on MNR, which can be determined by a simple equation. These results illustrate the importance of the saturation effect on the EPID and the need to select a correct value for NRP with respect to each “energy ‐ dose rate” combination. In practice, a rule of thumb has been suggested in order to quickly determine the optimum NRP to avoid detector saturation without degrading image quality.

## ACKNOWLEDGMENTS

We would like to thank Jean‐Yves Kristner and Christophe Lehobey for their helpful comments on the technology of both the aS500 detector and Varian LINACs, and Varian France for their support.
